# Not equal in the face of habitat change: closely related fishes differ in their ability to use predation-related information in degraded coral

**DOI:** 10.1098/rspb.2016.2758

**Published:** 2017-04-12

**Authors:** Maud C. O. Ferrari, Mark I. McCormick, Bridie J. M. Allan, Douglas P. Chivers

**Affiliations:** 1 Department of Biomedical Sciences, WCVM, University of Saskatchewan, 52 Campus Drive, Saskatoon, SK, Canada; 2 ARC Centre of Excellence for Coral Reef Studies, and Discipline of Marine Biology and Aquaculture, James Cook University, Townsville, Queensland, Australia; 3 Department of Biology, University of Saskatchewan, 112 Science Place, Saskatoon, Canada

**Keywords:** habitat degradation, coral reefs, coral bleaching, risk assessment, antipredator behaviour, alarm cues

## Abstract

Coral reefs are biodiversity hotpots that are under significant threat due to the degradation and death of hard corals. When obligate coral-dwelling species die, the remaining species must either move or adjust to the altered conditions. Our goal was to investigate the effect of coral degradation on the ability of coral reef fishes to assess their risk of predation using alarm cues from injured conspecifics. Here, we tested the ability of six closely related species of juvenile damselfish (Pomacentridae) to respond to risk cues in both live coral or dead-degraded coral environments. Of those six species, two are exclusively associated with live coral habitats, two are found mostly on dead-degraded coral rubble, while the last two are found in both habitat types. We found that the two live coral associates failed to respond appropriately to the cues in water from degraded habitats. In contrast, the cue response of the two rubble associates was unaffected in the same degraded habitat. Interestingly, we observed a mixed response from the species found in both habitat types, with one species displaying an appropriate cue response while the other did not. Our second experiment suggested that the lack of responses stemmed from deactivation of the alarm cues, rather than the inability of the species to smell. Habitat preference (live coral versus dead coral associates) and phylogeny are good candidates for future work aimed at predicting which species are affected by coral degradation. Our results point towards a surprising level of variation in the ability of congeneric species to fare in altered habitats and hence underscores the difficulty of predicting community change in degraded habitats.

## 1. Introduction

Habitat destruction is one of the major drivers of biodiversity loss worldwide [1,2]. While habitat loss has obvious immediate and high-impact ecological consequences, habitat degradation, in contrast, has slower, more subtle effects that are more difficult to detect [3]. Coral reefs are ecosystems that are at particular risk from habitat degradation. In these ecosystems, the health of corals are of prime importance as they represent ecosystem engineers, providing habitat to hundreds of animal and plant species [4]. Recent climatic changes, operating through an increased frequency of severe storms and ocean warming, have threatened the health and resilience of these ecosystems [5,6]. In fact, the Great Barrier Reef, the world's largest coral reef system, has recently experienced a period of ocean warming that may leave a tract of 1000 km of coral reefs experiencing 50–90% coral death [7]. While traditionally, biodiversity loss has been assessed through species extinctions, a few have argued that a missed component that often precedes those species extinctions are the alterations of ecological interactions in which these species are engaged [8,9]. Hence, studying changes in the way species interact in degraded coral reef ecosystems could provide insights into the resilience of the community in the face of environmental change.

Predation is a major force shaping communities, and has been ascribed a fundamental role in the promotion and maintenance of biodiversity. Due to the highly variable nature of predation, both in space and time, prey have evolved numerous ways to decrease their risk of capture. These adaptations include behavioural, morphological or life-history changes. Predation pressure, for instance, dictates where individuals forage, set up a territory and with whom they mate [10]. Some prey have predator-induced morphologies, such as protective spines or helmets that help reduce their rate of predation [11]. Others show these defensive morphologies from birth [12]. Prey with complex life histories can sometimes alter the timing of their transition from one stage to the next based on predation risk in either stage. For instance, predators capitalizing on eggs can induce prey to hatch earlier than those that are not exposed to predators [13]. Conversely, prey detecting predation risk in the next life stage can delay their transition in order to reach larger sizes before entering the next stage, thus increasing their chance of surviving [14]. Many more examples of phenotypic plasticity exist in response to predation [15]. Such alterations in prey defences have cascading effects, in the form of trait-mediated indirect interactions (TMII). It has been suggested that TMII are more regulatory in prey populations than traditional consumptive, density-mediated interactions [16]. Most TMII are inducible and expressed in a threat-sensitive manner, that is, they are expressed with a ‘strength’ that matches that of the risk perceived. Thus, in order to know when and how much to invest in antipredator defences, prey need to assess their risk of predation using cues from their environment.

In aquatic ecosystems, most prey rely on visual and chemical cues to assess risk [17]. Because visual cues are often limited by light availability and by highly complex habitats like coral reefs or kelp beds, and can be manipulated by predators via crypsis, it is not surprising that many aquatic prey have a strong reliance on chemical information to inform them about risk [18]. One of the most common classes of chemicals that aquatic prey use are injured conspecific cues, often referred to as alarm cues in fishes. These cues are present in the skin or tissues of conspecifics and are only released into the water column via mechanical damage to the skin or tissue, as would typically occur during a predator attack. As such, they represent a highly reliable indicator of risk, and are known to elicit immediate and dramatic antipredator responses in nearby conspecifics. These responses are highly conserved and documented in a wide variety of taxa, including corals, molluscs, crustaceans, fishes and larval amphibians [19]. The widespread use of these cues in aquatic systems illustrates the critical role they play for the survival and maintenance of populations. Indeed, these cues have been shown to elicit most trait-mediated indirect interactions discussed above, and many more, such as facilitating learned predator recognition [20]. Not surprisingly, the presence of these cues has been linked to increased prey survival during staged predator–prey encounters [21–23]. As such, these cues are considered a major source of information for prey.

Our present study aimed to assess the effect of coral degradation on the ability of coral reef fishes to detect and respond to injured conspecific cues. Previous work suggests that the Ambon damsel, *Pomacentrus amboinensis*, fails to respond to injured conspecific cues when the cues pass over a patch of degraded coral [24,25]. Recent research also suggests that this species is also unable to learn the identity of novel predators using chemical alarm cues, but a congeneric specialist of dead coral habitats was still able to use information contained within the alarm cues to identify threats [26]. This important ecological difference between closely related species begs the question of how widespread the negative effect of coral degradation on the use of chemical information is to coral reef fishes. Specifically, our first experiment investigated how widespread this phenomenon was, by testing six common and closely related damselfish species, sampling the species from a variety of habitats. Two species, *Pomacentrus moluccensis* and *Chromis viridis*, are found on healthy live corals (live coral associates). Two species, *P. chrysurus* and *P. nagasakiensis*, are commonly found on coral rubble (dead coral associates), while our last two species, *P. amboinensis* and *P. wardi*, are found on mixed habitat types (mixed associates). Each species was tested in both a live and dead coral environment for their response to their species’ injured conspecific cues or a heterospecific control. Predictions from our previous studies were that the alarm cue response of fish that are coral obligates may be most affected by coral degradation, while those more typically associated with dead and degraded habitats may have evolved a mechanism to circumvent the problem. A second experiment was performed to try and tease out the mechanism behind the results of experiment 1, specifically to test whether the lack of response of *P. amboinensis* in degraded coral water was due to the inactivation of the cues in that environment, or whether it was due to the inability of *P. amboinensis* to detect the cues via sensory interference.

## 2. Methods

### (a) Test species

Newly settlement-stage juvenile damselfish (five *Pomacentrus* species and one *Chromis* sp.—see electronic supplementary material for more details) were collected overnight using light traps moored in open water around Lizard Island (14′40° S, 145′28° E), in the northern Great Barrier Reef, Australia, in November 2015. The juveniles, sorted by species, were held in 20-l flow-through holding tanks and fed three-times a day with brine shrimp (*Artemia* nauplii). Apogonids (cardinalfish) were caught on the fringing reef using hand nets and fed fish pellets daily. They were used as heterospecific control (see below).

### (b) Experimental outline

The first experiment consisted of exposing six common species of damselfish juveniles to their injured conspecific cues or a heterospecific control (controlling for the smell of any fish; apogonid) in seawater flowing past either live or dead-degraded coral. The experiment followed a 6 × 2 × 2 completely randomized design.

The second experiment investigated possible mechanisms responsible for the loss of response of fish to alarm cues in degraded environments. We chose *P. amboinensis* and *P. nagasakiensis* juveniles for this experiment, as the former is affected while the latter appears unaffected by water that has been in contact with dead-degraded coral. The two species were maintained in two habitats (live or dead coral water), and were exposed to each other's injured cues or apogonid cues in a 2 × 2 × 3 completely randomized design. We predicted that if the absence of response of *P. amboinensis* is mediated via a deactivation of its alarm cues (hypothesis 1), then neither species should respond to *P. amboinensis* cues, while they should both respond to *P. nagasakiensis* cues. If, on the other hand, *P. amboinensis* cannot respond to its alarm cue due to sensory interference (hypothesis 2), then we predicted that *P. amboinensis* should not respond to the alarm cues from a closely related species, while *P. nagasakiensis* should respond to both cues. The protocol used to test the fish was identical for both experiments.

### (c) Experimental set-up

#### (i) Exposure phase

Groups of 10 juveniles were placed into 12-l plastic exposure tanks, which had flowing seawater from a header tank containing either live or dead coral. The header tank consisted of a 15-l Amundsen bucket containing either a piece (approx. 60 cm in circumference) of healthy, live *Pocillopora damicornis*, a hard bushy coral commonly found at our field site, or an equal sized piece of dead-degraded coral that was encrusted with algae. The header tanks were equipped with an airstone, and had constantly flowing fresh seawater at a rate of 1 l min^−1^ (one tank turnover every 12 min). The header tank was plumbed in such a way that allowed the overflow to enter the exposure tanks. Both coral types were changed daily. The fish were kept in the exposure tank for 48 h before the test phase.

#### (ii) Testing phase

Following the exposure phase, fish were moved individually into 5-l plastic tanks, equipped with a sand substrate, a moulded plastic replica of branched coral (15 cm high) serving as shelter, and an air stone, to which was attached a 1.5 m long injection hose. A 4 × 4 cm grid was drawn on the tank to facilitate data collection. Each test tank received flow-through water from a header tank containing live or dead coral, as described above. The difference was that the flow-through from the header tank was divided among five testing tanks. Each test tank thus received water at a rate of approximately 1 l/5 min (one tank turnover every 25 min). The fish were left to acclimate overnight and were tested the following day.

The bioassay followed established protocols [19] and is described in details in the electronic supplementary material. In short, the behaviours of each fish (number of feeding strikes and line crossed, as measures of feeding and activity) were observed for 3 min before and after the introduction of a stimulus (5 ml of alarm cues or apogonid cues). Reductions in feeding and activity are both well-established antipredator responses. We tested 244 fish (*n* = 10–11/treatment) in experiment 1 and 148 fish (*n* = 12–13/treatment) in experiment 2 (see electronic supplementary material for size). The observer was blind to the treatment and the order of treatments was randomized.

### (d) Statistical analysis

Given that feeding and activity are not independent variables, the two were analysed simultaneously using a MANOVA approach. Pre-stimulus data were first analysed to ensure there was no difference among treatment groups prior to stimulus injection. Pre- and post-stimulus data were then used to calculate a per cent change in behaviour [(post-pre)/pre] and the resulting variables were used in subsequent analyses. For experiment 1, both analyses (one for prestimulus baseline, one for behavioural change) were carried out using a three-way MANOVA, testing the effects of species, habitat type (dead versus live coral) and cue type (heterospecific versus conspecific cues) on behavioural responses. Subsequent two-way MANOVAs were used to explore possible interactions. For experiment 2, both analyses were performed using a three-way MANOVA, testing the effect of species (*P. amboinensis* versus *P. nagasakiensis*), coral type (live versus dead) and cue type (*P. amboinensis*, *P. nagasakiensis* or apogonid control). Subsequent two-way MANOVAs and Tukey HSD post-hoc comparisons were performed to explore interactions. For all tests, data met parametric assumptions.

## 3. Results

### (a) Experiment 1

The only factor explaining differences in pre-stimulus values was species (Pillai's Trace: *F*_10,440_ = 3.1, *p* = 0.001), indicating that fish from the same species exposed to different coral waters or given different cues did not differ in their baseline activity levels. No other factor was significant (all *p* > 0.4).

**Figure 1.**
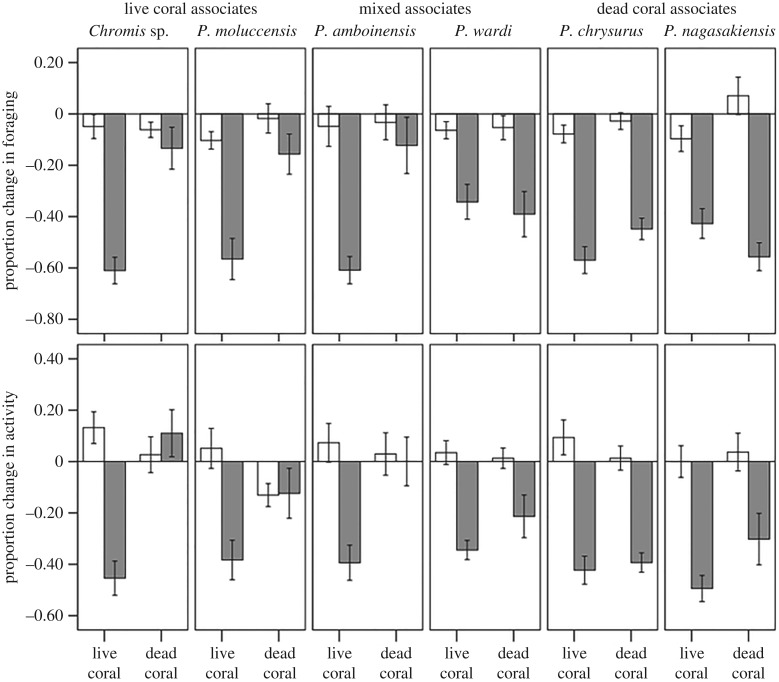
Mean (±s.e.) proportion change in the number of feeding strikes (top panel) and line crosses (bottom panel) for damselfish species that are typically associated with live coral only (live coral associates), degraded-dead coral only (dead coral associates) or species that settle in both types of habitat (mixed associates). Fish were maintained in water from either live or dead coral and exposed to cues from heterospecific apogonid (empty bars) or cues from injured conspecifics (solid bars) (*n* = 10–11/treatment).

Change in behaviour was influenced by a three-way interaction among species, cue and coral (Pillai's Trace: *F*_10,440_ = 3.6, *p* < 0.001, figure 1). Splitting the analysis between the two coral types revealed that, in live coral, all fishes responded to conspecific cues with a significant antipredator response (cue: Pillai's Trace: *F*_2,105_ = 152.4, *p* < 0.001). We failed to find an effect of species (Pillai's Trace: *F*_10,218_ = 1.3, *p* = 0.3) or an interaction between cue and species (Pillai's Trace: *F*_10,218_ = 1.3, *p* = 0.2), indicating that all species responded similarly to their respective alarm cues. On dead coral, however, a significant species by cue interaction (Pillai's Trace: *F*_10,222_ = 3.3, *p* = 0.001) indicated that species differed in their responses to alarm cues. Species found on live coral failed to respond to their alarm cues in dead coral (*P. moluccensis*: *F*_2,18_ = 1.3, *p* = 0.3; *Chromis*: *F*_2,18_ = 0.7, *p* = 0.5). Dead-degraded associates, on the other hand, maintained their response to alarm cues (*P. chrysurus*: *F*_2,17_ = 54, *p* < 0.001; *P. nagasakiensis*: *F*_2,18_ = 25, *p* < 0.001). Interestingly, species living in mixed habitats showed mixed responses, with *P. amboinensis* failing to respond to alarm cues (*F*_2,17_ = 0.2, *p* = 0.8), and *P. wardi* displaying a full antipredator response to the alarm cues (*F*_2,18_ = 5.7, *p* = 0.012).

### (b) Experiment 2

**Figure 2.**
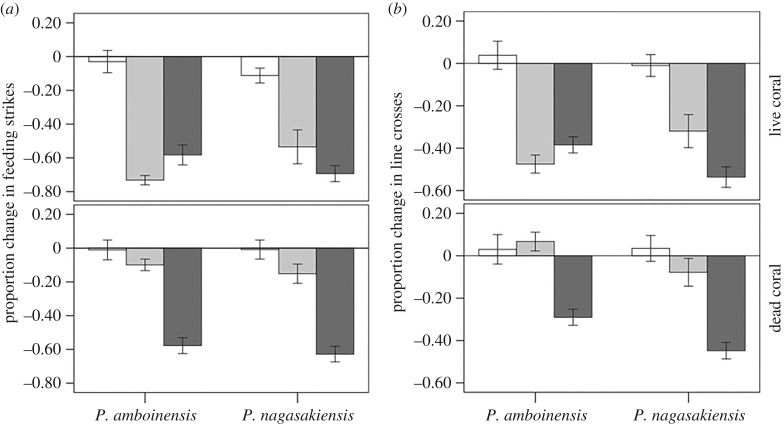
Mean (±s.e.) proportion change in the number of feeding strikes (*a*) and line crosses (*b*) for *P. amboinensis* or *P. nagasakiensis* tested in live coral water (top panels) or dead-degraded coral water (bottom panels). The fish were exposed to cues from distantly related apogonid (control, empty bars), cues from *P. amboinensis* (light grey bars) or cues from *P. nagasakiensis* (dark grey bars).

None of the treatment groups differed in their pre-stimulus baseline (Pillai's Trace: all *p* > 0.4). Change in behaviour was affected by an interaction between coral and test cue (Pillai's Trace: *F*_4,272_ = 12.1, *p* < 0.001, figure 2). Splitting the analysis by coral revealed that, in live coral, the responses of the fish were affected by an interaction between species and test cue (*F*_4,136_ = 2.8, *p* = 0.028). Specifically, both species displayed a significant antipredator response to the Pomacentrid alarm cues compared to the apogonid control (Tukey post-hoc comparisons: *P. amboinensis* versus apogonid: *p* < 0.001, *P. nagasakiensis* versus apogonid: *p* < 0.001 for both variables). However, each species responded to their own cues with a stronger intensity than to the one of the close relative (2 × 2 MANOVA: species × cue interaction: *F*_2,45_ = 4.3, *p* = 0.02). In dead coral, however, the pattern was different. Fish behaviour was affected by the type of cue they received (*F*_4,136_ = 20.1, *p* < 0.001), but there was no species by cue interaction (*F*_4,136_ = 0.7, *p* = 0.6). Both species responded with a significant antipredator response to *P. nagasakiensis* cues compared to apogonid cues (Tukey post-hoc comparisons: *p* < 0.001 for both variables), but failed to show a statistically significant response to *P. amboinensis* cues (*p* = 0.08 and *p* = 0.8 for feeding and activity respectively). For *P. nagasakiensis* cues, once again, the response from conspecifics was stronger than that of close relatives (*p* = 0.032).

## 4. Discussion

Coral degradation had dramatically different effects on the efficacy of alarm cues among closely related species. Of the six species tested, half maintained their response to injured conspecific cues in degraded corals, while the other half completely lost their ability to respond to the cues in the degraded habitat. This was a striking result, because although the composition of the active substance in the alarm cues is still unknown (and likely different for all species, since we do not see taxa-wide responses to a single cue), a number of studies found that these compounds were highly conserved among closely related species. For instance, several species of salmonid trout from a few genera can respond to each other's cues, although the strength of the response decreases with increased phylogenetic distance [27]. Similar results are found in other species, including damselfish [28]. Our findings emphasize that the interaction between the background olfactory landscape and chemical alarm cues is species specific and can differ between closely related fish.

Results suggest for *P. amboinensis* that the lack of response in a degraded environment may stem from a deactivation of the active component of their alarm cue. Indeed, while the expected cross-species response is intact in live coral environments, neither *P. amboinensis* nor *P. nagasakiensis* can respond to *P. amboinensis* cues in degraded coral. Interestingly, they can both respond to *P. nagasakiensis* cues in that same environment. That result suggests that *P. amboinensis* alarm cues are modified by the chemistry of water from degraded corals, while the same water does not affect *P. nagasakiensis* cues. We speculate that a chemical group nearby the active site of *P. amboinensis*’ cue either changes its conformation or binds with a water-borne compound, which blocks access to the active site, rendering the cue inactive. Another potential explanation for our results would be that the responses to species-specific alarm cues are mediated by species-specific receptors in the olfactory rosette, and that degraded coral water contains a compound that would block the receptors for *P. amboinensis* alarm cues in the rosette of both species. While technically possible, the information we have to date with regards to olfactory perception and neurobiology [29], the multi-compound nature of the alarm cues [30] and the principle of parsimony makes this alternative explanation less likely in our opinion. Exploring both these suppositions would require some advances in the field of vertebrate predation-related chemical ecology. The chemistry of these interactions remains sadly understudied [19,31].

Based on the previous findings, one of two scenarios, not necessarily mutually exclusive, may explain the pattern of responses we observed. First, the pattern of response follows that of the species’ habitat. Although we cannot test this hypothesis rigorously, our limited sample size (*n* = 4 species) provides preliminary evidence that habitat may be a good predictor of the impact of coral degradation on cue use. Both species typically associated with live coral lost the ability to respond appropriately to injured cues in a degraded habitat, while both species typically associated with rubble and dead coral maintained the appropriate cue response. This pattern was also found for *P. coelestis*, a dead coral associate [26]. Hence, the different sensitivity to degraded coral habitat could stem from local adaptation to microhabitat conditions, a hypothesis already present in the literature [32,33]. Rubble has always been a part of coral reef ecosystems. When corals die, their exoskeletons break apart and form rubble-dominated microhabitats, until new corals recruit and take over. Species that live in those habitats may have selected the habitats due to the combined benefits from lower competition and their unique ability to detect alarm cues, an ability that was inherently present or was selected for by predation-mediated natural selection.

The second scenario that could explain the pattern of response is phylogeny. Two relatively recent studies have defined the phylogenetic relationship among Pomacentridae [34,35]. Both of them have relationships among four of our species, but neither of those have tested *P. wardi*. From these two papers, we can make some general groupings: *Chromis viridis* is the most distantly related, *P. moluccensis* and *P. amboinensis* are sister species, and *P. nagasakiensis* and *P. chrysurus* are also closely related to each other. This phylogenetic pattern also matches our response patterns, with *P. nagasakiensis* and *P. chrysurus* maintaining their response to injured cues in degraded habitat, while *P. moluccensis* and *P. amboinensis* both lost their responses in the degraded habitat. Interestingly, according to Cooper *et al*. [34], *P. coelestis* is closely associated with *P. chrysurus*, and we see concordance in the response pattern of the two species in degraded habitats. It is difficult to conclude anything for the other species. Following the principle of parsimony, the change seen from a phylogenetic point of view may in turn explain the ecological segregation of the species based on their ability to use predation-related cues in degraded habitats.

For the species that lost their response to alarm cues, the ecological consequences are likely significant, with a potential decrease in all alarm cue-mediated indirect effects. The immediate effect of alarm cues is to warn nearby conspecifics of a recent predation attack. The increase in vigilance results in an immediate increase in survival over the next several minutes to hours [21]. However, alarm cues also facilitate learning and other lasting effects including investment in morphological defences [36,37]. Without these cues, these species will likely be much more vulnerable to predation. Many coral reef species, including our damselfishes, have a bipartite life history where pelagic larvae recruit to the reefs after 10–25 days and settle to become benthic juveniles. This transition is linked to a predation-mediated population bottleneck whereby 60–90% of juveniles are consumed within the first 2 days post-settlement [38]. There is immense selection for prey that can use alarm cues to reduce risk of predation. The loss of these cues by some members of the community will have far-reaching consequences for restructuring the community. For instance, the cross-species responses seen in our second experiment may indicate benefit for some species to associate with other species that can provide them with valuable public information regarding predation risk, such as would happen during cross-species social learning among guild members [39,40].

The present study provides a viable mechanism that explains the relatively rapid loss of species from systems where hard corals have died, despite the maintenance of topographic complexity for years after death. It provides a link between the expansion of dead-coral-dominated landscapes and their rapidly altered communities, such as those seen in the Caribbean [41]. A common pattern seen in many ecosystems is that generalist species that are able to survive in modified habitats have a competitive edge over specialists in the face of habitat change [42–44] and these species make up the new, modified community in altered environments. Our results provide evidence that some coral reef fish species are functionally more generalist than others, as demonstrated by their ability to use predation-mediated cues in both pristine and degraded coral environments. As such, we predict that these species will make up a higher proportion of the fish community in the reefs of the future, and that those that cannot adapt may slowly disappear.

## Supplementary Material

Method details

## Data accessibility

The data have been uploaded as electronic supplementary material.
