# Supplementary material for: Not equal in the face of habitat change: closely related fishes differ in their ability to use predation-related information in degraded coral
Source: Proc Biol Sci. 2017 Apr 12;284(1852):20162758. doi: 10.1098/rspb.2016.2758 (PMC5394659; doi:10.1098/rspb.2016.2758)
Supplement: Method details [file rspb20162758supp1.docx]

**Ferrari et al. Not equal in the face of habitat change: closely related fishes differ in their ability to use predation-related information in degraded coral.**

**Supplementary information**

**METHODS**

***Test species:*** To avoid phylogenetic confound, damselfishes (family Pomacentridae) were the focus of the present study. These are one of the most species-rich families of reef-associated fishes, with some 388 species within 28 genera, many of which live in tropical waters. They are variously omnivorous, but adults are usually planktivorous or herbivorous, and occupy a broad range of habitats with habitat associations that vary from obligate through to generalist. The present study focuses on five species from one of the most species-rich genera, *Pomacentrus*, together with another species *Chromis* sp., which was chosen for its obligate association with live hard coral as juveniles. All species have a dispersive larval phase of 14 to 27 days [[1](#_ENREF_1)] followed by settlement to juvenile habitats which is coincident with metamorphosis [[2](#_ENREF_2)]. At settlement, most damselfish species choose habitats with specific characteristics [[3](#_ENREF_3), [4](#_ENREF_4)] and move little from their initial settlement site [[5](#_ENREF_5)].

**Bioassay**: In each tank, we first introduced 2 mL of an *Artemia* solution containing ~100 *Artemia*/mL. After 3 min, we injected another 2 mL of food and started the 3-min pre-stimulus observation period. During that time, we recorded the number of feeding strikes of the fish (regardless if they were successful), and the number of lines crossed by the fish (a measure of activity; a line was counted when the entire body of the fish crossed the line). After this baseline observation period, we injected either 5 mL of a solution of conspecific alarm cues or a 5 mL solution of Apogonid injured cues. We then injected another 2 mL of food, and recorded the behaviour of the fish for another 3 min. The difference between the pre- and post-stimulus period indicated the response of the fish to the cues. A reduction in feeding and activity are both well-established antipredator responses [[6](#_ENREF_6)]. Conspecific injured cues were prepared by euthanizing a conspecific donor via cold shock, and making nine cuts on either side of its body. The body was then rinsed with 15 mL of seawater and this 15 mL solution was used fresh within 15 min of preparation, and yielded enough cues for three replicates (5 mL each). This concentration has previously been shown to elicit overt antipredator responses in damselfishes [[7](#_ENREF_7)]. The heterospecific cues were obtained by sacrificing a cardinalfish (*Apogonid* sp.) and preparing the cues in the same manner as previously described for conspecifics. The observer was blind to the treatment and the order of treatments was randomized.

**Fish size**:

**Experiment 1**: The mean total length ± SD of the fish tested were as follows: *P. amboinensis:* 13.0 ± 0.7 mm, *P. chrysurus*: 12.7 ± 0.6 mm, *P. moluccensis*: 12.4 ± 0.7 mm, *P. nagasakiensis*: 14.1 ± 0.7 mm, *P. wardi*: 13.5 ± 0.9 mm, *Chromis sp*.: 8.8 ± 1.4 mm. Fish placed in the live and dead coral treatment did not differ in size (all P>0.3), although fish from different species differed in their respective size

**Experiment 2**: Fish from the live and dead coral did not differ in size (P=0.8), but once again, *P. nagasakiensis* (15.0 ± 1.3 mm) were larger than *P. amboinensis* (13.1 ± 1.1 mm).

1. Wellington G.M., Victor B.C. 1989 Planktonic larval duration of one hundred species of Pacific and Atlantic damselfishes (Pomacentridae). *Marine Biology* 101, 557-567.

2. McCormick M.I., Makey L., Dufour V. 2002 Comparative study of metamorphosis in tropical reef fishes. *Marine Biology* 141(5), 841-853. (doi:10.1007/s00227-002-0883-9).

3. Ohman M.C., Munday P.L., Jones G.P., Caley M.J. 1998 Settlement strategies and distribution patterns of coral-reef fishes. *Journal of Experimental Marine Biology and Ecology* 225(2), 219-238.

4. McCormick M.I., Moore J.A.Y., Munday P.L. 2010 Influence of habitat degradation on fish replenishment. *Coral Reefs* 29(3), 537-546. (doi:10.1007/s00338-010-0620-7).

5. McCormick M.I., Weaver C.J. 2012 It pays to be pushy: intracohort interference competition between two reef fishes. *PloS one* 7(8), e42590.

6. Ferrari M.C.O., Wisenden B.D., Chivers D.P. 2010 Chemical ecology of predator-prey interactions in aquatic ecosystems: a review and prospectus. *Canadian Journal of Zoology* 88(7), 698-724.

7. Chivers D.P., McCormick M.I., Mitchell M.D., Ramasamy R.A., Ferrari M.C.O. 2014 Background level of risk determines how prey categorize predators and non-predators. *Proceedings of the Royal Society B: Biological Sciences* 281(1787), 20140355.
